# Applicants' Interview Experience of Family Medicine Residency Match: Reflections from a Quality Improvement Initiative at a Community Hospital

**DOI:** 10.7759/cureus.11054

**Published:** 2020-10-20

**Authors:** Abdul Waheed, Masooma S Rana, Muhammad A Rauf, Landen Green, Salma Green, Erum Azhar

**Affiliations:** 1 Family Medicine, Wellspan Good Samaritan Hospital, Lebanon, USA; 2 Medicine, Aga Khan University Hospital, Karachi, PAK; 3 Liver Transplant and Hepatobiliary Division, Section of Surgical Sciences, Vanderbilt University Medical Center, Nashville, USA; 4 Public Health Sciences, Penn State University College of Medicine, Hershey, USA; 5 Obstetrics and Gynecology, Maimonides Medical Center, Brooklyn, USA

**Keywords:** family medicine residency program, residency match, residency interview, community hospital, lcme-accredited, img, osteopathic schools

## Abstract

Background and objective

Both family medicine applicants and programs dedicate significant resources to the interview process, a time for both parties to make an impression on the other in an attempt to find their best match. Despite the importance of this process, little research has been completed to ensure the process efficiently addresses applicant preferences on interview day and the surrounding process. This study aimed to determine the factors influencing the family medicine applicant preferences regarding the pre-interview, interview, and post-interview ranking process.

Methods

The study method was a cross-sectional electronic survey utilizing anonymous questionnaires that assessed demographics, pre-interview, interview, post-interview ranking preference, and applicants' experiences applying to a community-based family medicine residency program after their interview for the 2020 application cycle.

Results

Out of the 106 family medicine applicants, 48 responded; 52.08% were males, 52.5% were married, 58.33% applicants were from the osteopathic medical school, 33.33% were from the allopathic Liaison Committee on Medical Education (LCME) non accredited medical school/international medical graduates (IMG's), and 8.33% were from the allopathic LCME accredited medical schools. Free hotel accommodation was not offered from half of the programs to 27.8% of the applicants in the 2020 match cycle (pre-pandemic). Respondents favored electronic means of scheduling interviews with a positive experience with the online self-scheduling Electronic Residency Application Service (ERAS) calendar. A significantly higher proportion of IMGs applied to a higher number of family medicine programs followed by the osteopathic applicants. There was no statistical difference found between osteopathic and allopathic applicants for the number of programs they got invited to; however, the difference was significant for osteopathic and allopathic LCME accredited applicants who interviewed and ranked programs in the range of 11-20 (62.96%, p=0.0013 and 66.67%, p=0.0018, respectively). The respondents' most important experiences were interviewing the program director, faculty members, and tour the hospital facility. When ranking programs, these family medicine applicants considered the strength of program training, the quality of current residents, and the program's geographic location as the top three most significant factors, with mean importance ratings of 5.08, 5.02, and 4.35, respectively. Applicants also considered how the current residents perceive the program director, prior teaching experience, and program diversity with mean importance ratings of 3.42, 2.89, and 2.09, respectively.

Conclusion

Although applicants' preferences for family medicine residency programs are similar to generally reported by The National Resident Matching Program (NRMP) surveys, some key differences do exist. The program leadership should consider these preferences from the candidates’ perspective for a successful match in family medicine residency on both sides.

## Introduction

The National Residency Matching Program (NRMP) can be an arduous process for potential residents. Residency programs evaluate and rank candidates based on a number of factors such as results of standardized exams like the United States Medical Licensing Examination (USMLE) or Comprehensive Osteopathic Medical Licensing Examination (COMLEX), letters of recommendations (LoR), research, and interviews. However, the NRMP ranking goes both ways, as the applicants also create rank lists for the programs they apply to. According to the 2019 NRMP Applicant Survey, all applicants considered the top four factors when ranking programs, including overall goodness of fit, desired geographic location, quality of the residents in the program, and their interview day experience [1]. 

Both applicants and residency programs devote significant time, resources, and expenses to the interview process, as this is an opportunity for each party to make an impression on the other.

On average, family medicine residency programs were found to spend $17,079 on interviewing annually and $234 per residency interview [2]. Applicants also have a great financial burden, as 64% reported spending at least $2,500 during the interview process [3]. Programs need to understand their applicants' needs for the interview day to ensure resources are utilized appropriately.

In 2019, 4,128 residency positions were available for family medicine, with 3,848 spots filled, with a fill rate of 93.2%. In 2020, the fill rate went down to 92.5% - the lowest rate since 2010. Additionally, fill rates for allopathic seniors reached historic lows in 2019 (39.2%) and 2020 (33.2%), resulting in more positions being taken up by osteopathic students and international medical graduates (IMGs) [4]. This diverse pool of applicants may have varied needs and preferences on interview day, such as travel, accommodation, etc. 

Currently, there are few studies concerning the family medicine residency interview process about the applicants' perspective and needs. To our knowledge, there have only been two papers published on the subject. Schneeweiss et al. were published in 1982; the match process has evolved significantly since then [5]. Woloski et al. recently conducted a study identifying applicants' interview day preferences at a family medicine university program [6]. 

There are currently no studies based at community hospitals or comparisons between center data and national NRMP data. Six-hundred and ninety-five family residency programs are registered with the NRMP, and out of those, 564 are community programs [7, 8]. Considering the diversity of both applicants and programs in the field, more research is needed in this area. 

This study is part of a quality assessment and improvement initiative for applicant experience during the residency interview season. It surveyed candidates applying to our community hospital, family medicine residency program on pre-interview, interview day experiences, and post-interview ranking preferences. This will serve the Family Medicine Match stakeholders to identify significant factors that influence the ranking process, specifically in community hospital-based family medicine residency programs.

## Materials and methods

The Family Medicine residency candidates applying to community hospital-based WellSpan Good Samaritan Hospital’s residency program in the 2020 application cycle were surveyed via an anonymous electronic survey questionnaire about their pre-interview and interview experiences. The survey required approximately five minutes to complete. A group of physician investigators knowledgeable in survey development adopted the survey used by Woloski et al. [6]. We estimated that at least 80% of the applicants who interviewed at our community programs would be able to answer the survey. Candidates were sent the electronic survey after their interviews at our community-based residency program. Questions included experiences related to the pre-interview, interview, post-interview, and ranking process. There were no questions that contained any personal identifiers. Given the type of survey, it was deemed exempt from full IRB review with determination as non-human subjects research (NHSR).

The questionnaire elicited information regarding demographics (four questions), pre-interview experience (eight questions), interview experience (nine questions), and post-interview/ranking questions (six questions). A multiple-choice response format was used for most questions. For most pre-interview experience questions, the response options were: all programs, nearly all programs, approximately three-fourths of the programs, half of the programs, and less than half of the programs. For interview experience questions, the options were rated on a scale from one to three: important, neutral/either way and not important/would rather keep it out. For the post-interview ranking questions, the options were rated on a scale from one to five about the importance of the ranking process for the applicant. A small number of the questions were open-ended (e.g., how many programs did you apply for this match season and how many programs invited you for the site visit/interview). 

Descriptive statistics (means, standard deviations, and proportions) were collected. The statistical software SAS 9.4 (Statistical Analysis System, SAS Institute, Cary, USA) was used for the categorical variables. The Chi-square statistics were calculated to analyze significant differences between different variables. A p-value of <0.05 was considered statistically significant. Statistical significance was also determined between different medical schools, applicant’s preferences, and application process. 

## Results

A total of 48 questionnaires were collected and analyzed from 106 family medicine residency applicants (response rate 45.2%) applying for the 2020 match. Table 1 describes the demographics of the respondents. Out of 48 respondents, 52.08% (n=25) were males whereas 43.75% (n=21) were females. About half of the respondents (52.5%, n=25) were married, with the remaining 47.92% (n=23) identifying themselves as singles. In terms of ethnicity, 35.42% (n=17) identified themselves as Asian, which was followed by Whites (31.25%, n=15), Hispanics/Latinos (10.42%, n=5), and Black (2.08%, n=1). The majority of the respondents attended osteopathic medical schools (58.33%, n=28). Among the allopathic medical schools, 33.33% (n=16) of the respondents were from LCME-non-accredited medical schools and international medical graduates (IMGs) and 8.33% (n=4) from LCME-accredited medical schools.

**Table 1:** Demographics of surveyed interviewed applicants from the 2019 application cycle

Characteristics	n	%
Gender		
Male	25	52.08%
Female	21	43.75%
Non-binary/other	1	2.08%
Prefer not to disclose	1	2.08%
Marital status		
Single	23	47.92%
Married	25	52.08%
Ethnicity		
White	15	31.25%
Asian	17	35.42%
Black	1	2.08%
Hispanic/Latino	5	10.42%
Other	7	14.58%
Prefer not to disclose	3	6.25%
Medical school status		
Allopathic, LCME-accredited	4	8.33%
Allopathic, LCME-non-accredited OR international medical school outside the USA	16	33.33%
Osteopathic	28	58.33%

**Figure 1:**
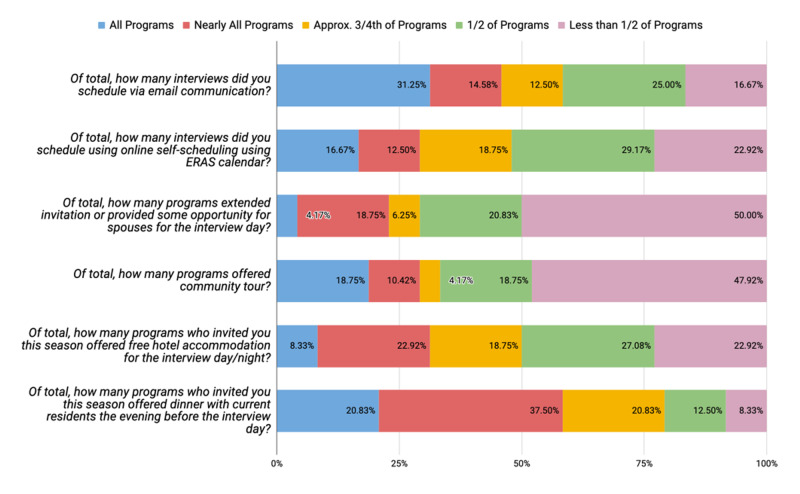
Distribution of applicants' responses regarding family medicine pre-interview experiences

Figure 1 shows the percentage of applicants' responses regarding the pre-interview experience. Nearly half of applicants stated that less than half of the programs they were invited to, offered a community tour (47.92%, n=23) and an extended invitation/opportunity for their spouses (50%, n=24) on interview day.

Many applicants were not offered free hotel accommodation from half of the programs they applied to 27.08% (n=13). A majority of applicants were offered dinner with current residents at the program before interview day from all (20.83%, n=10) or nearly all (37.50%, n=18) of the programs; though 87.50% (n=42) of applicants preferred to have dinner with current residents the evening before the interview (Table 2). More applicants were seen to schedule interviews with all or nearly all programs via email communication (45.8%, n=22) rather than the ERAS calendar (29.17%, n=14) (Figure 1). A majority of applicants had a positive experience with the online self-scheduling ERAS calendar, with half of the respondents recommending it highly (n=24) and 37.50% (n=18) thought it was “likable and good” (Table 2).

**Table 2:** Distribution of responses to additional questions regarding pre-interview and interview preferences ERAS - Electronic Residency Application Service

Questions	Responses	%
Please rate your experience of online self-scheduling using ERAS calendar	I prefer and recommended it highly	50.00%
	Likable and good	37.50%
	Difficult but doable	8.33%
	Difficult but some assistance from program preferred	2.08%
	Extremely difficult and would not recommend	2.08%
If given choice between the two, what is your preferred setting to interact with current residents?	Dinner the evening before the interview	87.50%
	Breakfast early morning the day of the interview	12.50%

**Figure 2:**
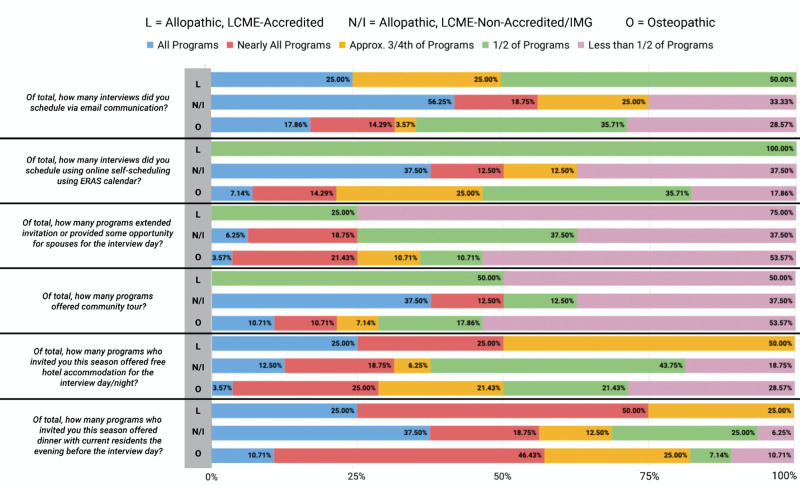
Distribution of applicants' responses regarding family medicine pre-interview experiences by medical school status L - allopathic LCME-accredited; N/I - allopathic LCME-non-accredited/international medical graduates; O - osteopathic; LCME - Liaison Committee on Medical Education

When the applicant's responses regarding family medicine pre-interview experience were analyzed according to the medical school status as shown in Figure 2, we noted that 56.25% of the applicants from allopathic LCME-non-accredited schools/IMG’s scheduled their interviews via email communication with all programs, whereas 25% of the applicants from allopathic LCME-accredited schools and 17.66% of the applicants from the osteopathic medical schools scheduled their interviews via email communications with all the programs. Online self-scheduling ERAS calendar was used by 37.5% of the applicants from the allopathic LCME-non-accredited schools/IMG’s for all programs, whereas 100% of the allopathic LCME-accredited and 35.71% of the applicants from the osteopathic medical schools scheduled their interviews using the online self-scheduling ERAS calendar for half of the programs.

Free hotel accommodation was provided for the interview day/night by all/nearly all programs to 50% of the applicants from the allopathic LCME-accredited medical schools, whereas it was provided to 30.75% of applicants from allopathic LCME-non-accredited schools/IMG’s and to 28.57% of the applicants from the osteopathic medical school. All programs/nearly all programs offered dinner with the current residents the evening before the interview day.

**Figure 3:**
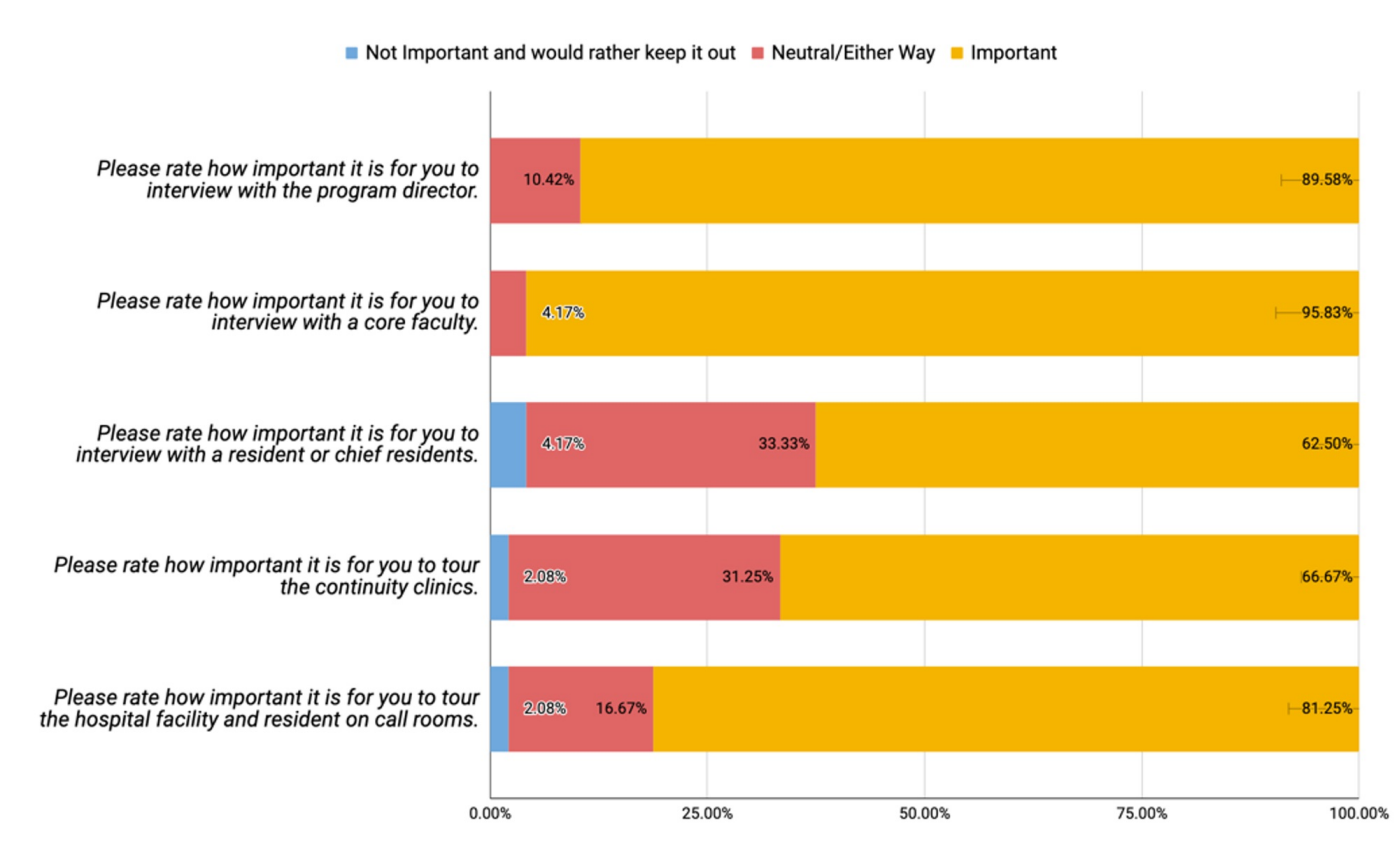
Distribution of applicants' responses regarding family medicine interview day experiences

In terms of the interview day experience, the majority of the applicants stated that it was important for them to interview with the core faculty and program director - 95.83% and 89.58%, respectively. Concerning the importance, the next rankings were about the hospital facility and resident on-call rooms tours (81.25%), continuity clinic tour (66.67%), and interview with a resident or chief resident (62.50%) during the interview day as shown in Figure 3.

**Figure 4:**
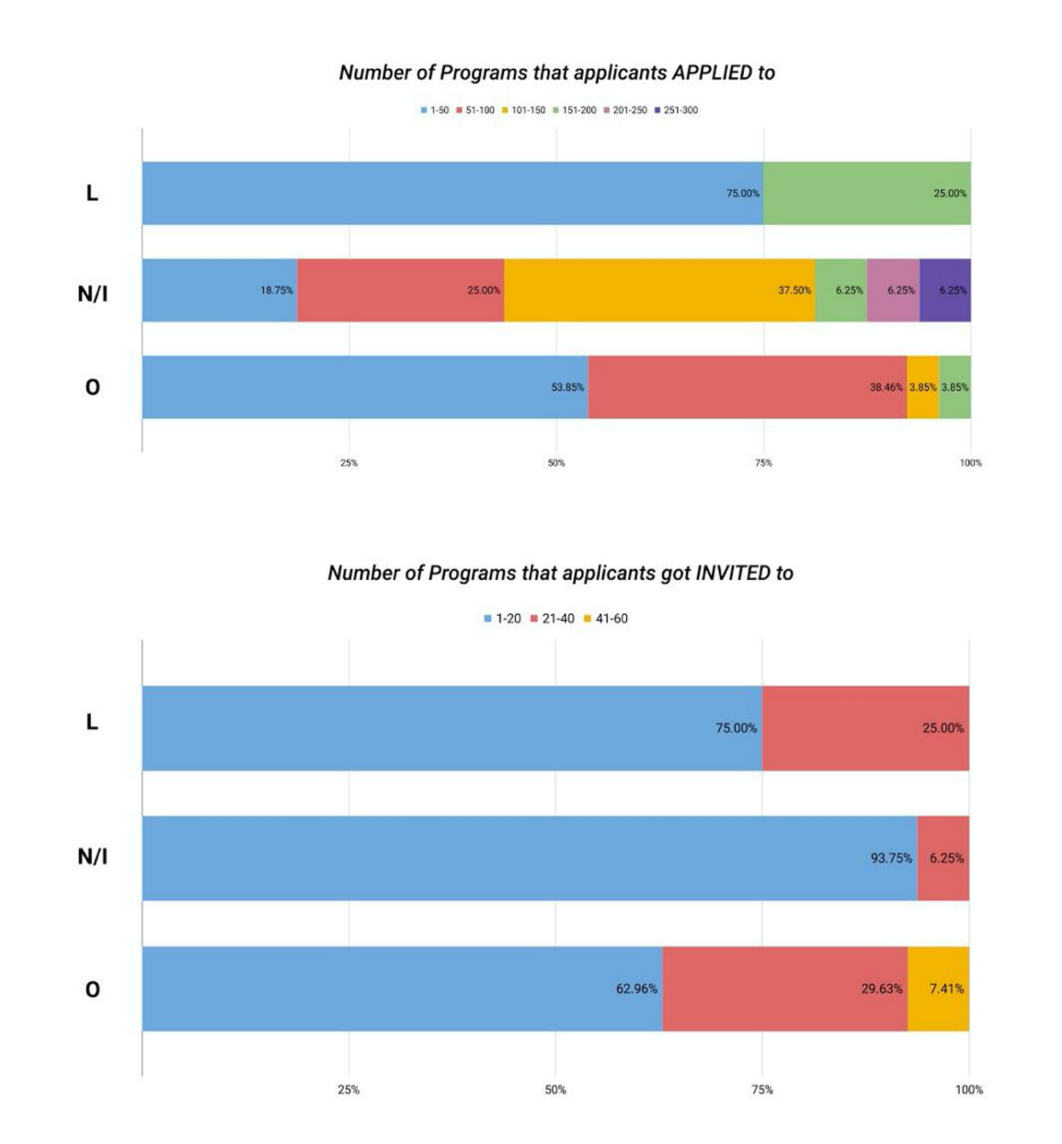
Number of programs applicants applied and were invited by school type L - allopathic LCME-accredited; N/I - allopathic LCME-non-accredited/international medical graduates; O - osteopathic; LCME - Liaison Committee on Medical Education

Out of the allopathic LCME-non-accredited/IMGs applicants, 37.5% applied to 101-150 programs; however, 53.5 % of the osteopathic applicants and 75% of the applicants from allopathic LCME-accredited medical schools applied to the 1-50 programs as shown in Figure 4.

**Figure 5:**
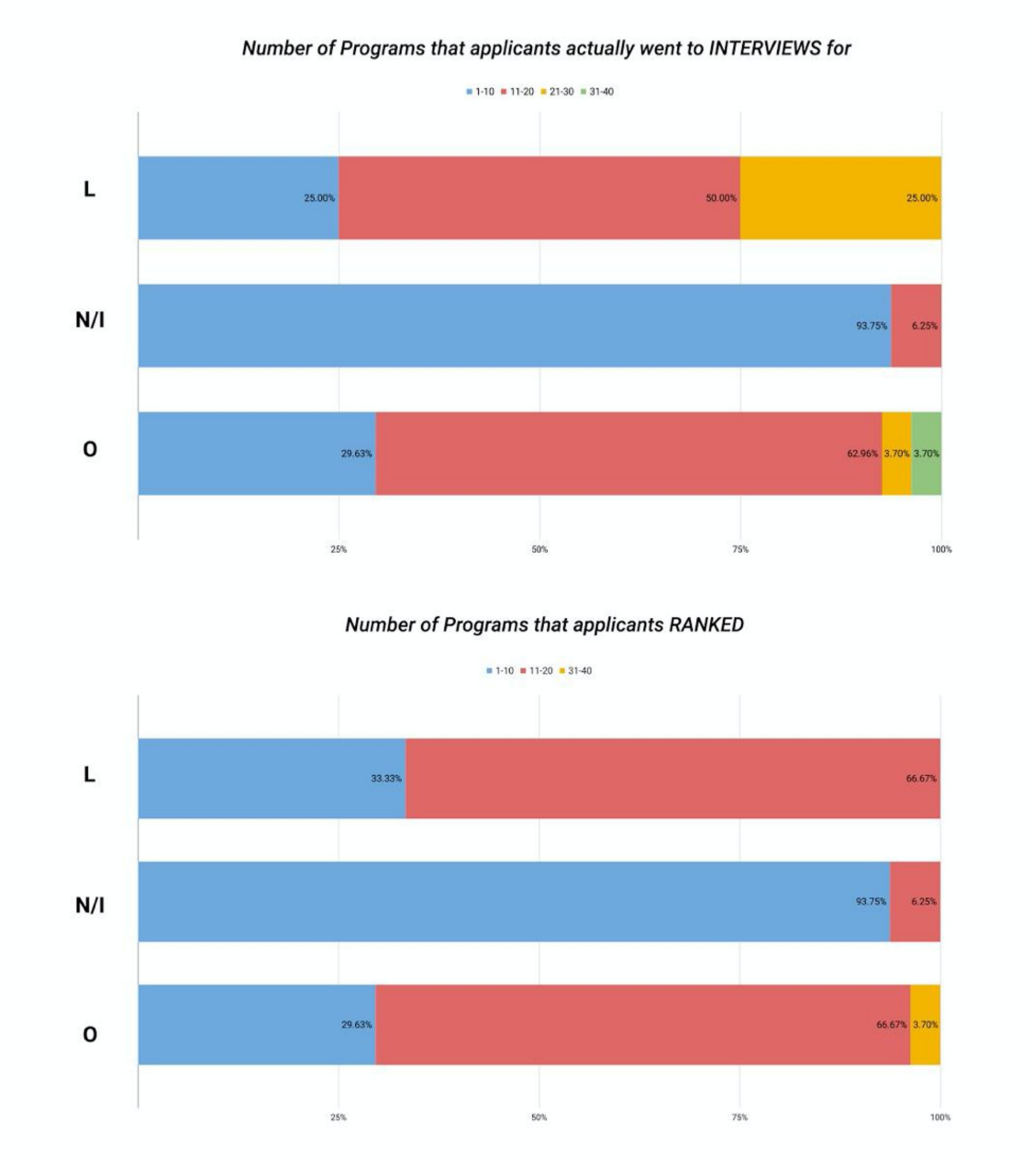
Distribution of the number of programs applicants went to interviews and were ranked by applicants' school type L - allopathic LCME-accredited; N/I - allopathic LCME-non-accredited/international medical graduates; O - osteopathic; LCME - Liaison Committee on Medical Education

The results on the number of programs applied to by the applicants from different medical school categories were statistically significant, with a p-value of 0.0302. The number of applied programs was statistically significantly different (p=0.018) between applicants from allopathic medical schools and applicants from osteopathic medical schools. A significantly higher proportion of IMGs applied to a higher number of programs followed by the osteopathic applicants (p=0.0302). We did not find any significant difference between applicants from allopathic LCME-non-accredited/IMG’s (75%), LCME-accredited (93.75%), and osteopathic schools (62.96%) in the range of 1-20 programs that they got invited to for interview (p=0.25). However, we did find significant statistical significance (p=0.0013) between applicants from allopathic LCME-non-accredited/IMG’S (25%), LCME-accredited (93.75%), and osteopathic schools (29.63%) who were interviewed for 1-10 programs. Many osteopathic applicants (62.96%) were interviewed for 11-20 programs. Ranking of the programs in the range of 1-10 was statistically significantly different (p=0.0018) among applicants from allopathic LCME non-accredited/IMG’s (33.33%), LCME-accredited (93.75%), and osteopathic schools (29.63%). A considerable percentage (66.67%) of applicants from osteopathic and allopathic LCME-accredited schools ranked programs in the range of 11-20, as shown in Figure 5.

**Figure 6:**
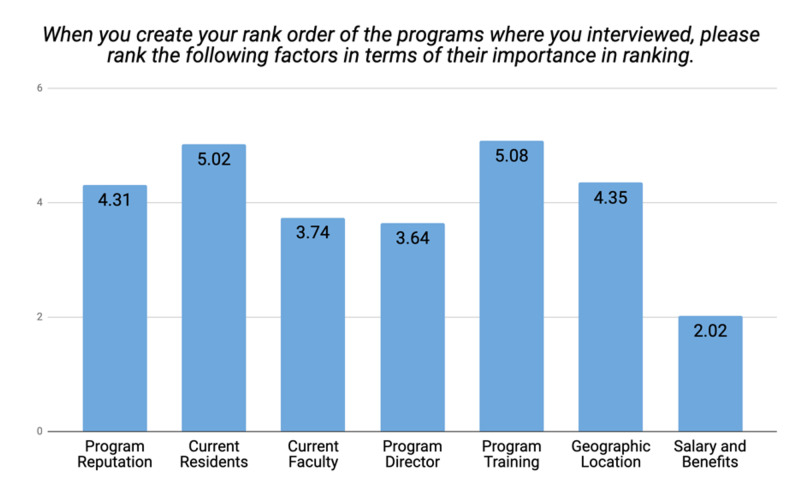
Applicants' mean importance rating of the programs' factors

While ranking the programs, applicants considered the strength of program training, current residents' diversity and background, and geographic location of the program as the top three most significant factors, with mean importance ratings of 5.08, 5.02, and 4.35, respectively (Figure 6).

The training program's strength was considered an important factor while ranking by 58.33% of applicants from osteopathic medical school, 33.33% of applicants from the allopathic LCME-non-accredited/IMG’s and 8.33% of the applicants from allopathic LCME-accredited medical schools.

It was also interesting to find out that the strength of the training program was nearly equally highly ranked by all ethnicities, then the geographic location of the program was given the highest score by the White applicants with a mean score of 5.53 as compared to applicants with other ethnic backgrounds (p<0.0001). Current residents with their diversity and happiness in the program were highly evaluated by the Black applicant with a mean score of 6, followed by applicants who preferred not to disclose their ethnicity with a mean score of 5.67 and Asians with a mean score of 5.63 (p=0.0001). The diverse background and experience of the current faculty were ranked highest by Hispanic/Latino applicants (mean score of 4.20) followed by Asians and Black respondents with mean scores of 4.18 and 4, respectively (p=0.0003). The program director's teaching experience and diversity factor were scored highest by applicants who marked their ethnicity as "others'' and Asians with mean scores of 4.67 and 3.82, respectively (p=0.0013).

**Figure 7:**
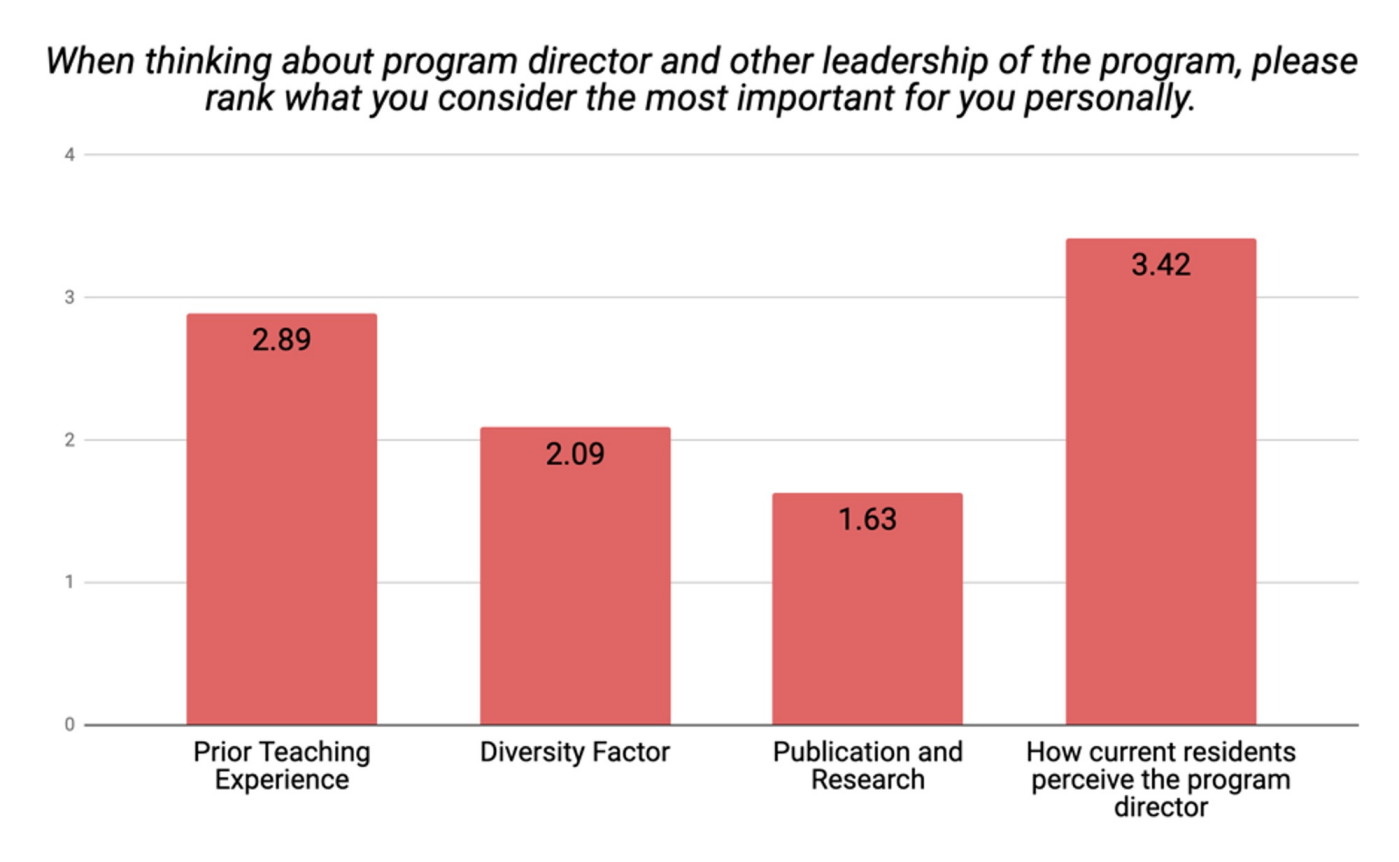
Applicants' mean ratings on the importance of program leadership factors

Considering the program's leadership, applicants took into account how the current residents perceive the program director, prior teaching experience, and diversity factor with mean importance ratings of 3.42, 2.89, and 2.09, respectively (Figure 7).

Interestingly, when these results were analyzed according to various ethnicities of the applicants, Black applicant evaluated highly how the current residents perceive the program directors with a mean score of 4, followed by applicants who marked their ethnicity as "other" or preferred not to disclose their ethnicity with mean scores of 3.71 and 3.67, respectively, compared to their White applicants with a mean score of 3.60 (p<0.0001). Similarly, the diversity factor (ethnicity/race/cultural background/religion and gender) of the leadership was evaluated higher by Hispanic/Latino applicants with a mean score of 3.40, followed by applicants who marked their ethnicity as "other" and Asians with mean scores of 2.50 and 2.06, respectively (p<0.0001). The program director's prior teaching experience was ranked highest by those applicants who preferred not to disclose their ethnicity, followed by White applicants with mean scores of 3.33 and 3.20, respectively (p<0.0001).

## Discussion

This study examined the residency applicants' preferences and experiences during the family medicine residency match application, specifically the experiences during the pre-interview process and at the interview day, interview scheduling, and post-interview ranking.

For the past eleven years, the number of family medicine residency positions offered and filled has increased steadily. However, it has been postulated that this is only a consequence of an increase in graduates' number, not an increase in interest [9]. In the 2020 NRMP match, family medicine offered 13.7% and filled 13.4% of the total positions, with an overall fill rate of 92.5%. As previously mentioned, fill rates for family medicine have never been lower, especially for allopathic seniors, with only 8.6% matching to family medicine residency positions in the 2020 NRMP match, with a fill rate of 33.2% down from 45.1% in 2018 [4, 10]. The "America Needs More Family Doctors: 25x2030 collaborative" launched by the American Academy of Family Physicians calls for 25% of all allopathic and osteopathic US seniors to choose family medicine as a career by 2030; currently, it is 12.6% [4]. This trend is deeply concerning for the future of primary care, with shortages in family physicians directly affecting rural and disadvantaged populations, which the primary care physicians are more likely to care for as compared to primary care internists and pediatricians [11].

Despite minimal numbers for allopathic seniors, there are increases for every other applicant type. In 2020, the number of osteopathic US seniors matching to family medicine reached a historical high, 23.4% (versus 19.5 % in the 2019 match), with 104 more applicants matching in NRMP than 2019 combined NRMP and American Osteopathic Association's (AOA) match. It could be to the fact that most osteopathic institutions have a strong orientation towards primary care, while a number of allopathic schools simply see it as another specialty [10]. The last two decades have seen growth in the number of applicants for the first-year residency spot in the NRMP match. There seems to be rapid growth in international medical graduates and osteopathic graduates. Additionally, 42 more US IMGs and 36 more foreign IMGs matched to family medicine in the 2020 NRMP match compared to the 2019 NRMP match [4]. The number of increasing osteopathic medical students participating in the match has also been partly due to the increase in the number of new osteopathic schools [12]. Both these populations should be targeted to fill the gaps in primary care, especially osteopathic US seniors.

This study shows that the osteopathic and IMG applicants apply to a higher number of programs than LCME-accredited allopathic medical students. There is not much published data on this, given that 2020 was the first match where students who graduated from osteopathic schools competed directly with all other applicants in NRMP. Before this, they had a separate National Match Program (NMP). With participation in regular matches and integration of osteopathic programs into the Accreditation Council for Graduate Medical Education (ACGME), many osteopathic schools have anecdotally been reported to advise their students to apply and interview "widely" to increase their chances. Weissbart and colleagues discussed such behaviors in the light of game theory and prisoner's dilemma for the Urology Residency Match specifically, but it is widely applicable to all NRMP [13].

International medical graduates in residency recruitment are primarily stigmatized due to their limited clinical experience in the US as well as the impact on a program's reputation if applicants deemed less competitive are accepted [14]. Anecdotally, programs with more IMGs are considered less competitive, although there are no actual parameters or objective standards to show than. On the contrary, there is some evidence that IMGs work at par or sometimes better than their local LCME-accredited counterparts. For example, the 2017 mortality analysis of older patients who were treated in the US hospitals by internists found slightly lower mortality rates among international medical graduates after adjusting for patient and physician characteristics and fixed hospital effects [15]. However, many international medical graduates apply to far more programs to secure a successful match compared to their US graduates. These current study results are consistent with that. It is also evident that the students and graduates from the local AOA Commission on Osteopathic College Accreditation (COCA) accredited osteopathic schools also hang out in a boat similar to IMGs, as noted above.

The sociodemographic characteristics of our sample closely represent the target population similar to the results of the biennial 2018 NRMP Program Directors survey, especially when compared with those family medicine programs which typically interview and rank each applicant group (US senior, US graduate, osteopathic physician, Canadian, Fifth pathway, IMG and Non-US IMG). ​​​​​Percentage of programs that interview and rank each applicant type was 99% US seniors, 94% osteopathic physicians, 77% US IMGs, 44 % non-US IMGs, and 42% Canadian applicants. Our survey had 58.33% representation from osteopathic medical school, 33.33% in combined allopathic, LCME-non-accredited, or international medical school, and 8.33% from allopathic LCME-accredited programs [9]. We found in our study that a large number of applicants were not offered complimentary hotel accommodations from the programs they applied to. Programs should consider the cost vs. benefit of accommodating certain applicants' needs by understanding who is applying to their program and their preferences. This, however, will not be a major factor in Match 2021 as programs and applicants move towards virtual interviews during the COVID-19 pandemic.

The Accreditation Council for Graduate Medical Education (ACGME) requires all residency programs to have diversity. They state: "CPR 1.C. The program, in partnership with its Sponsoring Institution, must engage in practices that focus on mission-driven ongoing, systematic recruitment and retention of a diverse workforce inclusive of residents, fellows (if present), faculty members, senior administrative staff members, and other relevant members of its academic community" [16]. Jarman et al. conducted a study to assess underrepresented minority (URiM) applicants in general surgery residency programs as well as to determine the impact of URiM faculty and residents on URiM applicants' selection for interview or match. URiMs were defined as applicants who self-identified as black/African American, Hispanic/Latino/of Spanish origin, and American Indian/Alaskan Native/Native Hawaiian/Pacific Islander-Samoan. They found that there was no difference in selection to interview for URiM (OR: 0.83; 95% CI: 0.54 to 1.28, per 10% increase in faculty diversity) or non-URiM applicants (OR: 0.68; 95% CI: 0.57 to 0.81) as the diversity of the faculty increased. Interestingly, greater representation of the URiM among the current residents did not affect the likelihood of being selected for an interview for URiM (OR: 1.20; 95% CI: 0.90 to 1.61) vs. non-URiM applicants (OR: 1.28; 95% CI: 1.13 to 1.45). Thus, those programs with a greater proportion of URiM core faculty or residents did not select a greater proportion of URiM applicants for interviews. Hence, diversity-conscious recruitment of racially and ethnically diverse trainees and faculty requires further research, which can help find effective recruitment strategies that cater the diverse applicant pools from all ethnicities and races and diverse underserved US populations [17]. Although it might seem that URiM-conscious or generally diversity-conscious recruitment may require intervention at the pre-interview selection process, the current study sheds light on the preferences of applicants belonging to diverse backgrounds. For example, diverse faculty and program leadership were more preferred by Non-white applicants as compared to whites/Caucasian Americans. The geographic location, on the other hand, was more preferred by white applicants as compared to non-white applicants. Some of these results are intuitive and may provide impetus to change to some programs. The programs cannot change their geographic location, but they could look into diversifying the complement of faculty and leadership parallel to resident recruitment efforts.

Figure 6 shows that the family medicine applicants rated the strength of program training, current residents' diversity and background, and geographic location of the program as the top three most significant factors, with mean importance ratings of 5.08, 5.02, and 4.35, respectively.

Data are scarce regarding the preferences of applicants to family medicine residency programs. An old study exploring the characteristics of the interview day that were preferred by the medical students applying to family practice residency programs showed that interviews and meetings with residents informally were considered as most helpful for applicants; this was followed by interviews with the program director or faculty. Kikano et al. surveyed the program directors to explore the markers of successful recruitment of students to family practice residency programs and demonstrated high-quality current residents and faculty as well as the current residents' positive feeling about the program to be statistically significantly associated with a successful match (p<0.01) [18]. Woloski et al. conducted an anonymous questionnaire-based survey from applicants applying to a university program for family medicine residency to explore the applicant's preferences and demonstrated that more applicants preferred the use of an electronic form of communication. For applicants, it was important to interview with both the program director and current resident; also, they emphasized the importance of covering hotel accommodation as well as the inclusion of spouses or significant others [6]. The results of Woloski et al. are consistent with this current study.

Although the strength of the training program was nearly equally highly ranked by all ethnicities, the program's geographic location was given the highest score by the white applicants with a mean score of 5.53 compared to applicants with other ethnic backgrounds (p-value <0.0001). Current residents with their diversity and happiness in the program were highly ranked by the black applicant with a score of 6, followed by applicants who preferred not to disclose their ethnicity with a mean score of 5.67 and Asians with a mean score of 5.63 (p=0.0001). The current faculty's diverse background and experience were ranked highest by Hispanic/Latino (a mean score of 4.20), followed by Asians and black applicants with mean scores of 4.18 and 4, respectively (p=0.0003). The program director's teaching experience and diversity factor were scored highest by applicants who marked their ethnicity as "other'' and Asians with mean scores of 4.67 and 3.82, respectively (p=0.0013). Although there is limited data directly addressing the above racial disparities for family medicine programs, there is general literature suggesting that applicants from underrepresented minority backgrounds are more likely to rank programs favorably with greater gender and racial diversity. Ku et al. conducted a study on nineteen residency programs at the Sandford University School of Medicine and concluded that for the women and minority applicants, "perceived" program diversity is an important factor in making their decision for program ranking in surgical training [19]. Furthermore, residency programs with a greater diversity may be more attractive to URiM applicants as these programs may show a perception of being more committed to providing care for the underserved population [19-21]. There are significant barriers in achieving diversity in residency programs, including the limited understanding of how underrepresented applicants select and rank the residency programs as well as a perceived lack of diversity among the residents and faculty of residency programs, which may be interpreted by URiM applicants as a lack of support and commitment by the residency program and institution for any outcome related to diversity [19, 21, 22]. Effective recruitment would start with an evidence-based strategy for recruiting a competent and diverse physician workforce and identifying the applicants' preferences, especially the under-represented applicants [21, 23].

Overall, the current study shows results regarding family medicine resident applicants' key factors of ranking are similar to the 2019 NRMP applicant survey. This includes the strength of program training, quality of current residents, and the geographic location of the program as the top applicant priorities overall. The NRMP's top factors for both allopathic and independent (osteopathic and IMG) family medicine applicants for ranking programs were nearly identical to each other, with "overall goodness of fit", interview day experience, and quality of residents in the program being the top three factors for both [1].

This study also reflects that the individual family medicine residency programs can develop their own quality improvement projects for the applicants' recruitment process to ensure appropriate resource utilization for conscious recruitment and successful match. Future studies with a larger sample size involving multiple family medicine programs with representation from both community and university programs would be needed to understand the preferences of the family medicine applicants so that resources are directed to individual applicant types and eradicating any racial disparities.

This study has both strengths and weaknesses. This study was performed as a quality assurance (QA) initiative. The results showed significant insights that are worthy of sharing with the general audience. This study was carried out at a single community hospital within a single specialty of family medicine, which remains a significant limitation. Additionally, the sample size was small, with a response rate of nearly half of what we anticipated from applicants. The demographics of sampled applicants are comparable to the general 2019 NRMP applicant survey. This does allow some generalizability of the results to other programs. However, with smaller sample size and with a diverse population, it was difficult to perform significant statistical sub-analysis.

## Conclusions

Although the preferences of applicants to family medicine residency programs are similar to generally reported by NRMP surveys, some key differences also exist. The program leadership should consider these preferences from the applicants' perspective for a successful match on both sides. The matching process is the beginning of a relationship between the matched applicant and the program, not merely the filling of a potential spot. Residency programs should adopt a diversity-conscious or, more specifically, URiM-conscious approach to accommodate the rapidly diversifying pool of future applicants, embracing equity and inclusion, and adapt the interview day to the applicants' specific preferences. This will ensure the appropriate distribution of costs and recruitment of highly qualified applicants. In the future, multicenter studies should be conducted to understand further the needs of URiM candidates applying to family medicine.

## References

[1] National Resident Matching Program, Data Release and Research Committee (2019). Results of the 2019 NRMP Applicant Survey by Preferred Specialty and Applicant Type. Research Committee: Results of the.

[2] Nilsen K, Callaway P, Phillips JP, Walling A (2019). How much do family medicine residency programs spend on resident recruitment? A CERA study. Fam Med.

[3] Fogel HA, Liskutin TE, Wu K, Nystrom L, Martin B, Schiff A (2018). The economic burden of residency interviews on applicants. Iowa Orthop J.

[4] Family Medicine 2020 (2020). 2020 Match® Results for Family Medicine. https://www.aafp.org/students-residents/residency-program-directors/national-resident-matching-program-results.html.

[5] Schneeweiss R, Bergman J, Clayton J (1982). Characteristics of the residency interview process preferred by medical student applicants. J Fam Pract.

[6] Woloski JR, Schlegel D (2018). Surveying applicants to improve the family medicine residency interview day. PRiMER.

[7] (2020). ACGME - accreditation data system. https://apps.acgme.org/ads/Public/Reports/Report/1.

[8] (2020). FREIDA™ residency program database. https://freida.ama-assn.org/Freida/.

[9] Phillips JP, Wendling A, Bentley A, Marsee R, Morley CP (2019). Trends in us medical school contributions to the family physician workforce: 2018 update from the american academy of family physicians. Fam Med.

[10] National Resident Matching Program, Data Release and Research Committee (2018). Results of the 2018 NRMP Program Director Survey. https://www.nrmp.org/wp-content/uploads/2018/07/NRMP-2018-Program-Director-Survey-for-WWW.pdf.

[11] Xierali IM (2018). Distributional differences between family physicians and general internists. J Health Care Poor Underserved.

[12] Shannon SC, Teitelbaum HS (2009). The status and future of osteopathic medical education in the United States. Acad Med.

[13] Weissbart SJ, Hall SJ, Fultz BR, Stock JA (2013). The urology match as a prisoner's dilemma: a game theory perspective. Urology.

[14] Lin TY (1973). The psychiatric educational back- ground of foreign medical graduates. Am J Psychiatry.

[15] Tsugawa Y, Jena AB, Orav EJ, Jha AK (2017). Quality of care delivered by general internists in US hospitals who graduated from foreign versus US medical schools: observational study. Br Med J.

[16] (2020). Common Program Requirements (Residency) Sections I-V Table of Implementation Dates. https://www.acgme.org/Portals/0/PFAssets/ProgramRequirements/CPRResidencyImplementationTable.pdf.

[17] Jarman BT, Borgert AJ, Kallies KJ (2020). Underrepresented minorities in general surgery residency: analysis of interviewed applicants, residents, and core teaching faculty. J Am Coll Surg.

[18] Kikano GE, Galazka SS, Flocke SA, Saffran E, Zyzanski SJ (1994). Markers of successful recruitment of students to family practice residency programs. Fam Med.

[19] Ku MC, Li YE, Prober C, Valantine H, Girod SC (2011). Decisions, decisions: how program diversity influences residency program choice. J Am Coll Surg.

[20] Richmon EE, Ku BS, Cole AG (2019). Advocating for underrepresented applicants to psychiatry: perceptives on recruitment.. Am J Psychiatry Resid J.

[21] Pierre JM, Mahr F, Carter A, Madaan V (2017). Underrepresented in medicine recruitment: rationale, challenges, and strategies for increasing diversity in psychiatry residency programs. Acad Psychiatry.

[22] Saha S, Guiton G, Wimmers PF, Wilkerson L (2008). Student body racial and ethnic composition and diversity-related outcomes in US med- ical schools. JAMA.

[23] Saha S (2014). Taking diversity seriously: the merits of increasing minority representation in medicine. JAMA Intern Med.

